# Factors associated with discontinuation of biologics in patients with inflammatory arthritis in remission: data from the BIOBADASER registry

**DOI:** 10.1186/s13075-023-03045-3

**Published:** 2023-05-22

**Authors:** Marta Valero, Carlos Sánchez-Piedra, Mercedes Freire, María Colazo, Noemí Busquets, Erardo Meriño-Ibarra, Carlos Rodríguez-Lozano, Sara Manrique, Cristina Campos, Fernando Sánchez-Alonso, Isabel Castrejón

**Affiliations:** 1 Servicio de Reumatología, Hospital Universitario Ramón Y CajalInstituto Ramón Y Cajal de Investigación Sanitaria (IRYCIS), Carretera de Colmenar Viejo Km: 9,100, 28034 Madrid, Spain; 2 Health Technology Assessment Agency of Carlos III Institute of Health (ISCIII), Madrid, Spain; 3 Department of Rheumatology, Complejo Hospitalario Universitario A Coruña, A Coruña, Spain; 4 Department of Rheumatology, Hospital Universitario de Burgos, Burgos, Spain; 5 Department of Rheumatology, Hospital General de Granollers, Granollers, Barcelona Spain; 6 Department of Rheumatology, Hospital Universitario Miguel Servet, Saragossa, Spain; 7 Department of Rheumatology, Hospital Universitario de Gran Canaria Doctor Negrín, Las Palmas de Gran Canaria, Spain; 8 Department of Rheumatology, Hospital Universitario Regional de Málaga (Hospital Carlos Haya), Málaga, Spain; 9 Department of Rheumatology, Hospital General Universitario de Valencia, Valencia, Spain; 10 Research Unit of Spanish Society of Rheumatology, Madrid, Spain; 11 Department of Rheumatology, Hospital General Universitario Gregorio MarañónInstituto de Investigación Sanitaria Gregorio Marañón (IiSGM), Universidad Complutense de Madrid, Madrid, Spain

**Keywords:** Remission, Biologic DMARD, Discontinuation, Real-world evidence

## Abstract

**Background:**

The objectives of this study were to assess the discontinuation of biologic therapy in patients who achieve remission and identify predictors of discontinuation of biologics in patients with inflammatory arthritis in remission.

**Methods:**

An observational retrospective study from the BIOBADASER registry comprising adult patients diagnosed with rheumatoid arthritis (RA), ankylosing spondylitis (AS), or psoriatic arthritis (PsA) and receiving 1 or 2 biological disease-modifying drugs (bDMARDs) between October 1999 and April 2021. Patients were followed yearly after initiation of therapy or until discontinuation of treatment. Reasons for discontinuation were collected. Patients who discontinued bDMARDs because of remission as defined by the attending clinician were studied. Predictors of discontinuation were explored using multivariable regression models.

**Results:**

The study population comprised 3,366 patients taking 1 or 2 bDMARDs. Biologics were discontinued owing to remission by 80 patients (2.4%): 30 with RA (1.7%), 18 with AS (2.4%), and 32 with PsA (3.9%). The factors associated with a higher probability of discontinuation on remission were shorter disease duration (OR: 0.95; 95% CI: 0.91–0.99), no concomitant use of classic DMARDs (OR: 0.56; 95% CI: 0.34–0.92), and longer usage of the previous bDMARD (before the decision to discontinue biological therapy) (OR: 1.01; 95% CI: 1.01–1.02); in contrast, smoking status (OR: 2.48; 95% CI: 1.21–5.08) was associated with a lower probability. In patients with RA, positive ACPA was associated with a lower probability of discontinuation (OR: 0.11; 95% CI: 0.02–0.53).

**Conclusions:**

Discontinuation of bDMARDs in patients who achieve remission is uncommon in routine clinical care. Smoking and positive ACPA in RA patients were associated with a lower probability of treatment discontinuation because of clinical remission.

**Supplementary Information:**

The online version contains supplementary material available at 10.1186/s13075-023-03045-3.

## Background

The therapeutic management of inflammatory arthritis, including rheumatoid arthritis (RA) and spondyloarthritis, has changed substantially in the last 2 decades thanks to the introduction of early treatment and the emergence of new biological disease-modifying drugs (bDMARDs) and targeted synthetic DMARDs (tsDMARDs). The main objectives of therapy are to achieve remission, or at least minimum disease activity, and thus ensure optimal control of symptoms and progression, to preserve physical function and quality of life, and to minimize toxicity according to comorbid conditions [1, 2].

Current evidence and consensus are sufficient to recommend an optimization strategy once remission is achieved. This can take the form of dose reduction or increased periodicity of bDMARDs in order to avoid adverse events while maintaining efficacy [3–5]. Evidence supporting the discontinuation of biologics is not consistent. Nevertheless, this strategy can be used in clinical practice [6].

When biologic therapy is discontinued, the patient may experience a flare, although disease activity is controlled in most cases with the reintroduction of the bDMARD. The percentage of patients who achieve bDMARD-free remission is small and varies considerably with factors such as the disease itself, time since diagnosis, and previous strategy [7–10].

A better understanding of potential factors that could help identify patients who are more likely to achieve remission and maintain it without therapy could help rheumatologists make clinical decisions. More and more studies are evaluating treatment-free remission. According to previous reports on RA, factors associated with an increased chance of remission are the absence of autoantibodies (rheumatoid factor [RF] and anti-citrullinated peptide antibody [ACPA]), younger age, male sex, shorter disease duration, rapid response to treatment, improved physical function, absence of the shared epitope, and disease activity level. However, data for most of these factors are inconsistent [7, 11].

Few studies have evaluated potential predictors of treatment-free remission in spondyloarthritis [12, 13]. According to some reports, the factors associated with improved response to therapy include younger age, more severe inflammation, shorter disease duration, male sex, non-smoking status, and rapid response to treatment, although it remains unclear whether these factors are associated with treatment-free remission [14–16].

Most of the previous data are obtained from clinical trials in which therapy is prescribed according to a protocol, with results not necessarily reflecting real life. This study aims to assess factors associated with treatment discontinuation on remission as defined by the physician.

## Methods

### Study design

We performed a multicenter retrospective analysis in a real-world setting. Information was obtained from BIOBADASER, a national prospective registry of patients with rheumatic diseases treated with bDMARDs, including biosimilars, and tsDMARDs, either with approved or off-label indications. BIOBADASER has been collecting patient data continuously since October 1999. The cut-off for data collection in the present study was April 2021.

### Population

This nested cohort comprised adult patients (≥ 18 years) from the BIODASER registry. Patients with RA, ankylosing spondylitis (AS), or psoriatic arthritis (PsA) as defined by their clinician and with a ts/bDMARD as either first- or second-line treatments were included. The non-inclusion of patients in the third line or later enabled a more specific case definition and a more homogeneous sample. In addition, biologic-free remission in patients starting with the third line is even more infrequent.

Our group of interest included patients who discontinued treatment on achieving remission (assessed by the attending rheumatologist). Patients receiving ongoing treatment (> 1 year of follow-up) or who had discontinued treatment for other reasons were selected as a comparison group. We performed a sensitivity analysis, in which discontinuation due to remission was defined as the suspension of a b/tsDMARD for at least 6 months.

The possible heterogeneity of the comparison group was evaluated using a new sensitivity analysis, in which patients who had discontinued owing to lack of efficacy were excluded from the comparison group. Only patients receiving ongoing treatment or who had discontinued treatment for reasons other than remission or lack of efficacy were selected as a comparison group.

### Variables

The variables analyzed were as follows: (1) demographic data, including sex, age, diagnosis, disease duration, and comorbidities; (2) data on treatment, such as type of biologic and duration of medication use, reason for discontinuation; (3) disease activity indexes, namely, the 28-joint Disease Activity Score (DAS28) for RA and PsA and the Bath Ankylosing Spondylitis Disease Activity Index (BASDAI) for patients with spondyloarthritis at initiation of the previous b/tsDMARD; (4) other disease-specific variables, namely, RF and ACPA for patients with RA and HLA-B27 for patients with spondyloarthritis. Patients were classified according to their disease activity status as DAS28 ≥ 3.2 or BASDAI ≥ 4 depending on their diagnostic group. The outcome of interest (treatment interruption and reason for interruption) did not present missing values. No imputations were considered for missing values in independent variables.

### Statistical analysis

The results of the descriptive analyses were presented as frequencies and percentages for qualitative variables and as median or mean values for continuous variables. Patients were classified into 2 groups according to whether remission resulted in discontinuation of the biologic. Inter-group comparisons were made using the *t* test to analyze differences between means; the chi-square test was used for comparisons between proportions. Non-parametric tests (i.e., Kruskal–Wallis) were used in the case of data that were not normally distributed. Normality in distribution was tested using graphical and numerical methods. We used a bivariable logistic regression model to assess factors potentially associated with the discontinuation of the biologic due to remission. For the multivariable model, variables with *p* < 0.15 in the bivariable analysis and variables of clinical interest were selected. Indeed, we selected variables in order to avoid collinearity, maintain the confounders, and ensure optimal interpretation of the results and their subsequent clinical applicability. Backward elimination was then used to select the final model. All statistical tests were 2-sided; *p* values < 0.05 were considered to indicate a statistically significant result. All analyses were performed using Stata version 13.1 (Stata Corp., College Station, TX, USA; 2013).

### Ethical considerations

This study was performed in accordance with the Declaration of Helsinki (1975), as revised in 2013. All patients signed an informed consent document before inclusion in the register. All data in the BIOBADASER 3.0 registry are anonymized. The study was approved by the Ethics Committee of Hospital Clínic, Barcelona (approval code FER-ADA-2015–01).

## Results

From a total of 3366 eligible patients who received at least 1 biologic as first- or second-line treatment, 80 (2.4%) discontinued on achieving remission based on the criteria of their rheumatologist: 30 patients with RA (1.7%), 18 with AS (2.4%), and 32 with PsA (3.9%). Patients’ characteristics are shown in Table 1.

**Table 1:** Demographic and clinical characteristics of patients with inflammatory arthritis who discontinued therapy according to clinical remission

		Total	Disc. due to rem	No disc. due to rem	*P* value**
*N*		3366	80	3286	
Median duration of follow-up (years) (IQR:p25–p75)		3.04 (1.16–6.95)	5.57 (3.73–8.89)	2.98 (1.16–6.87)	< 0.001
Age, years mean (SD)		51.8 (13.1)	49.9 (15.1)	51.8 (13.0)	0.202
Female sex, *n* (%)		2133 (63.4)	43.0 (53.8)	2090 (63.6)	0.071
Age at diagnosis, mean (SD)		43.2 (13.7)	43.3 (14.4)	43.2 (13.6)	0.950
Disease duration, years, mean (SD)		8.6 (8.4)	6.6 (4.7)	8.6 (8.4)	0.036
Smoker, *n* (%)	Never	331 (9.8)	68.0 (85.0)	2165 (65.9)	0.004
	Current	2233 (66.3)	9.0 (11.3)	695 (21.2)	
	Previous	704 (20.9)	2.0 (2.5)	329 (10.0)	
Charlson Comorbidity Index, mean (SD)		2.1 (1.4)	1.8 (1.5)	2.1 (1.4)	0.143
Previous biologic DMARD, *n* (%)	First-line	1955 (58.1)	47.0 (58.8)	1908 (58.1)	0.902
	Second-line	1411 (41.9)	33.0 (41.3)	1378 (41.9)	
Concomitant DMARD	MTX	1414 (63.7)	25.0 (44.6)	1389 (64.2)	0.003
	LFN	639 (33.9)	11.0 (22.0)	628 (34.2)	0.071
	SSZ	174 (10.6)	5.0 (10.0)	169 (10.6)	0.893
Time on treatment with the previous biologic agent, mean (SD)		24.9 (34.3)	50.2 (40.0)	24.3 (33.9)	< 0.001
Biologic discontinued, *n* (%)	TNF-i	2575 (76.5)	72 (90.0)	2503 (76.2)	0.304
	IL6-i	197 (5.9)	3 (3.8)	194 (5.9)	
	CD20-i	189 (5.6)	2 (2.5)	187 (5.7)	
	JAK-i	70 (2.1)	0 (0)	70 (2.1)	
	IL17-i	109 (3.2)	0 (0)	109 (3.3)	
	IL12-23-i	28 (0.8)	0 (0)	28 (0.9)	
	Apremilast	57 (1.7)	0 (0)	57 (1.7)	
	Abatacept	134 (4.0)	3 (3.8)	131 (4.0)	
RF-positive, *n* (%)		828 (24.6)	9 (11.3)	819 (24.9)	0.002
ACPA-positive, *n* (%)		758 (36.8)	3 (8.6)	755 (37.3)	0.112
HLA-B27-positive,* n* (%)		671 (19.9)	18 (22.5)	653 (19.9)	0.203
Moderate-high disease activity at initiation of biologics^a^		457 (17.8)	9 (17.0)	448 (17.8)	0.874

Data are shown as mean (SD), except for categorical variables, which are shown as total number (percentage)

*Disc* Discontinuation, *Rem* Remission, *MTX* Methotrexate, *LFN* Leflunomide, *SSZ* Sulfasalazine, *i* inhibitor, *RF*, rheumatoid factor; *ACPA*, anti-citrullinated peptide antibody

^a^Moderate-high disease activity was defined as DAS28 ≥ 3.2 or BASDAI ≥ 4, depending on the disease

^**^p-value for possible differences between the group of patients who suspend treatment owing to remission and the rest of the population (patients receiving ongoing treatment or who had discontinued treatment for other reasons)

The descriptive analysis revealed that patients who did not discontinue bDMARDs have a longer disease course (mean of 8.6 years vs. 6.6 years; *p* = 0.036). This group included a higher percentage of smokers (21.2% vs. 11.3%) and former smokers (10.0% vs. 2.5%), with a significant inverse association between smoking and the possibility of achieving remission (*p* = 0.004). Of the patients who managed to discontinue treatment because of remission, 44.6% were receiving concomitant methotrexate, compared with 64.2% in the group that continued treatment (*p* = 0.003). Time to treatment with the most recent b/tsDMARD was greater in patients who discontinued treatment on achieving remission than in those who did not (mean, 40 months vs. 33.9 months; *p* < 0.001).

Supplementary Table 1 details the results by disease. The descriptive analysis only revealed differences with respect to comorbid conditions measured according to the Charlson comorbidity index in AS. Among patients with RA, 72% of those who discontinued treatment on achieving remission had a positive ACPA titer, compared with 23% of those whose treatment was discontinued because of remission (*p* = 0.002).

Supplementary Table 2 shows descriptive results from a sensitivity analysis, excluding patients who had discontinued owing to lack of efficacy.

### Factors related to discontinuation of biologics on achieving remission

The multivariable analysis of predictive factors related to discontinuation on achieving clinical remission revealed that those associated with a lower probability of discontinuation were longer disease duration (OR: 0.95; 95% CI: 0.91–0.99), concomitant use of a conventional systemic DMARD (csDMARD) (OR: 0.56; 95% CI: 0.34–0.92), and smoking (OR for non-smokers: 2.48; 95% CI: 1.21–5.08). Longer duration of the previous bDMARD was associated with a higher probability of discontinuation (OR: 1.01; 95% CI: 1.01–1.02) (Table 2).

**Table 2:** Regression models for all the diseases together

		Bivariable model			Multivariable model		
		Crude OR	95% CI	*P* value	Adjusted OR	95% CI	*P* value
Female sex		0.67	(0.43–1.04)	0.073	0.68	(0.40–1.13)	0.138
Age at onset		0.99	(0.97–1.01)	0.202	1.01	(0.99–1.03)	0.489
Disease (ref RA)	AS	1.48	(0.82–2.68)	0.19	0.98	(0.52–2.31)	0.802
	PsA	2.40	(1.45–3.97)	0.001	1.96	(1.10–3.50)	0.023
TNF-i (ref remaining treatments)		2.82	(1.35–5.87)	0.006	1.43	(0.63–3.25)	0.397
Smoking (ref smoker)	Non-smoker	2.43	(1.20–4.89)	0.013	2.48	(1.21–5.08)	0.013
	Ex-smoker	0.47	(0.10–2.18)	0.335	0.63	(0.13–2.97)	0.556
Second line of treatment (vs first line)		0.97	(0.62–1.53)	0.902	1.52	(0.93–2.49)	0.098
Disease duration		0.97	(0.93–1.00)	0.037	0.95	(0.91–0.99)	0.006
Concomitant csDMARD		0.60	(0.39–0.94)	0.026	0.56	(0.34–0.92)	0.021
Moderate-high activity^a^		1.44	(0.38–5.39)	0.591			
Time on treatment with the previous biologic agent		1.01	(1.01–1.02)	< 0.001	1.01	(1.01–1.02)	< 0.001
Year of discontinuation of treatment		0.98	(0.93–1.02)	0.309	0.96	(0.91–1.02)	0.157

*OR* Odds ratio, *95% CI* 95% confidence interval, *TNF-i* Tumor necrosis factor alfa inhibitor, *RA* Rheumatoid arthritis, *AS* Ankylosing spondylitis, *PsA* Psoriatic arthritis, *csDMARD* conventional synthetic DMARD

^a^High activity: DAS28 ≥ 3.2 or BASDAI ≥ 4

Predictive factors were also analyzed by diagnosis group (Table 3). In patients with RA, a positive ACPA titer was associated with a lower probability of discontinuation because of remission (OR: 0.11; 95% CI: 0.02–0.53). Older patients with AS were less likely to discontinue therapy because of remission (OR: 0.95; 95% CI: 0.91–0.99). We did not confirm an association between diagnosis and sex, smoking status, or DMARDs, probably because of the smaller sample size when analyzing by disease.

**Table 3:** Regression analysis by disease

		RA			AS			PsA		
		OR	95% CI	*P* value	OR	95% CI	*P* value	OR	95% CI	*P* value
Female sex		1.40	(0.16–12.54)	0.765	0.52	(0.16–1.68)	0.276	0.64	(0.30–1.33)	0.241
Age		1.02	(0.97–1.06)	0.450	0.95	(0.91–0.99)	0.016	1.00	(0.97–1.04)	0.822
TNF-i		0.41	(0.08–1.97)	0.265	-	-	-	-	-	-
Smoking (ref smoker)	Non-smoker	2.53	(0.29–22.19)	0.401	0.34	(0.09–1.22)	0.099	1.66	(0.57–5.08)	0.371
	Ex-smoker	1.43	(0.08–25.78)	0.807				0.67	(0.07–6.23)	0.723
Order of treatment		3.74	(0.95–14.71)	0.06	0.84	(0.25–2.74)	0.767	1.53	(0.69–3.42)	0.298
Disease duration		0.98	(0.90–1.07)	0.649	0.94	(0.86–1.04)	0.228	0.99	(0.93–1.05)	0.778
ACPA (ref negative)	Positive	0.11	(0.02–0.53)	0.006	-	-	-	-	-	-
HLA B27 (ref negative)	Positive	-	-	-	0.51	(0.15–1.78)	0.290	1.16	(0.39–3.47)	0.786
Time on treatment with the previous biologic agent		1.03	(1.02–1.05)	< 0.001				1.01	(1.00–1.02)	0.005
Year of discontinuation of treatment		0.88	(0.73–1.06)	0.178	0.90	(0.80–1.02)	0.11	0.99	(0.90–1.08)	0.787

*OR* Odds ratio, *95% CI* 95% confidence interval, *TNF-i* Tumor necrosis factor alfa inhibitor, *RA* Rheumatoid arthritis, *AS* Ankylosing spondylitis, *PsA* Psoriatic arthritis, *RF* Rheumatoid factor, *ACPA* Anti-citrullinated peptide antibody

There were no ex-smokers for AS

We performed an additional analysis of the 53 patients who remained in remission 6 months after discontinuation (Table 4 and Supplementary Tables 3 and 4). This analysis showed that the factors associated with a lower probability of remaining in remission 6 months after discontinuation were female sex (OR: 0.52; 95% CI: 0.28–0.97) and longer disease duration (OR: 0.94; 95% CI: 0.89–0.98). In contrast, the factors associated with a greater probability of achieving discontinuation were non-smoking status (OR: 4.34; 95% CI: 1.53–12.28) and longer duration of the previous bDMARD (OR: 1.01; 95% CI: 1.01–1.02).

**Table 4:** Sensitivity analysis: results of the regression analysis taking into account discontinuation of biologics on achieving remission, when this was maintained for at least 6 months

		Bivariable			Multivariable		
**Variable**		OR	95% CI	*P*-value	OR	95% CI	*P*-value
**Female sex**		0.59	(0.35–1.02)	0.060	0.52	(0.28–0.97)	0.039
**TNF-i**		1.76	(0.83–3.75)	0.143	0.99	(0.41–2.37)	0.977
**Disease (ref RA)**	**AS**	1.35	(0.66–2.74)	0.407	1.11	(0.45–2.73)	0.820
	**PsA**	1.94	(1.04–3.60)	0.036	1.51	(0.74–3.07)	0.256
**Smoking (ref smoker)**	**Non-smoker**	3.77	(1.35–10.51)	0.011	4.34	(1.53–12.28)	0.006
	**Ex-smoker**	0.53	(0.06–4.74)	0.596	0.64	(0.07–5.83)	0.691
**Second-line treatment**		1.06	(0.61–1.84)	0.831	1.65	(0.91–3.00)	0.100
**Corticosteroids**		0.70	(0.37–1.33)	0.273	-	-	-
**Concomitant treatment**		0.87	(0.50–1.50)	0.619	0.89	(0.48–1.63)	0.707
**Moderate-high activity**^a^		0.38	(0.12–1.25)	0.113	-	-	-
**Age at diagnosis**		1.00	(0.98–1.02)	0.663	-	-	-
**Age at onset**		0.99	(0.97–1.01)	0.425	1.01	(0.98–1.03)	0.530
**Disease duration**		0.96	(0.92–1.00)	0.052	0.94	(0.89–0.98)	0.010
**Charlson Comorbidity Index**		0.96	(0.79–1.17)	0.677	-	-	-
**Time in treatment**		1.01	(1.01–1.02)	0.001	1.01	(1.01–1.02)	< 0.001
**Year of discontinuation of treatment**		1.00	(0.94–1.06)	0.991	0.99	(0.92–1.06)	0.712

OR Odds ratio, *95% CI* 95% confidence interval, *TNF-i* Tumor necrosis factor alfa inhibitor, *RA* Rheumatoid arthritis, *AS* Ankylosing spondylitis, *PsA* Psoriatic arthritis, *RF* Rheumatoid factor, *ACPA* Anti-citrullinated peptide antibody

^a^Moderate-high activity was defined as DAS28 ≥ 3.2 or BASDAI ≥ 4 depending on the disease

The main factors influencing discontinuation due to remission were similar in the analysis of patients receiving ongoing treatment or who had discontinued treatment for reasons other than remission or lack of efficacy as the comparison group (Supplementary Tables 5 and 6).

## Discussion

Using a national registry database, we found discontinuation of biologics to be infrequent in patients with inflammatory arthritis who achieve remission in routine clinical care. The factors associated with the discontinuation of biologics in patients who achieve remission included smoking status, disease duration, use of csDMARDs, and duration of treatment with biologics. In addition, women are less likely to achieve biologic-free remission. Moreover, age in AS and absence of ACPA in RA were associated with remission.

To our knowledge, the subject of discontinuation has been explored in routine care, although mainly for RA patients and frequently when treatment has been tapered. Figures for discontinuation of treatment on achieving remission vary between studies and depend on differences in the definition of and approach used to measure the variable of interest, as well as the different study populations selected [17, 18]. In another study from Spain, less than 5% of patients discontinued biological therapy [19]. In a recent review, Verstappen et al. [7] showed that in patients with RA, DMARD-free remission was observed in 5–24% of clinical trials and in 23–28% of observational studies, although the patients included generally had early-onset RA, with specific therapeutic strategies, and often treatment only with csDMARDs.

In the case of AS, data from real-world studies are scarce, and it is more difficult to compare results between them owing to the heterogeneous nature of the disease itself and to the fact that most of the studies include optimization strategies. Even so, data on discontinuation because of remission are in agreement with our results, reporting low percentages: 1% in the DANBIO cohort [20] and 2% in the PULSAR cohort of American veterans with AS [21]. Similarly, the few available real-world data on PsA show a very low frequency of discontinuation of bDMARDs on achieving remission, e.g., the real-world study of 3 hospitals in Italy (1.7%) [22] or the recent study on the DESIR cohort of patients who initiated TNF inhibitors during the first 4 years of follow-up (only 1 of 182 patients) [13].

In our study, smoking was one of the factors associated with a poorer outcome overall, and this finding was confirmed in the sensitivity analysis at 6 months. The absence of statistical significance in the analysis by disease is probably due to the lower sample size. The association between smoking and poorer health outcomes in inflammatory disease is widely demonstrated in the literature [23–26], although data on a possible association with successful discontinuation of DMARDs are discordant or inconclusive [7, 11, 27].

As for differences by sex, the supplementary analysis at 6 months revealed a negative association between female sex and the possibility of not receiving bDMARDs at 6 months. Previous studies have shown that female sex is associated with a lower probability of remission, as seen in the meta-analysis of Hamann et al. [28] in patients with RA. Systematic reviews have revealed contradictory findings for RA. For example, Schlager et al. [11] found that a greater percentage of women did not experience a relapse after discontinuation, and Tweehuysen et al. [27] found no evidence of an association between sex and possible discontinuation of bDMARDs. Our data are in line with those of most of the studies that revealed differences and agree with studies that showed differences by sex for achieving or maintaining remission or low disease activity without DMARDs in patients with RA and AS [7, 13, 23, 29–32].

Consistent with our findings, several authors have also identified longer disease course as being negatively associated with achieving bDMARD-free remission [11, 13, 27, 28, 31–35]. We found that concomitant csDMARD therapy was negatively associated with discontinuation on achieving remission, although this association was not confirmed in the analysis at 6 months. Previous studies, such as that by Tweehuysen et al. [27] in patients with RA or the CRESPA study in patients with peripheral AS [36], found no positive or negative association for this factor, although in studies that did in fact show differences in maintenance of bDMARD-free remission, taking csDMARDs was positively associated with this possibility [31, 32]. In clinical practice, discontinuation of bDMARDs may be easier for patients whose disease progresses well and who do not therefore require a csDMARD, but for whom this could prove useful for ensuring bDMARD-free treatment in the long term.

While we found that patients in the remission group were younger, this factor was not significantly associated with discontinuation overall. However, this association was significant in patients with AS when the analysis was replicated by disease, consistent with other studies showing that younger age is a predictor of response [37, 38].

In our cohort, patients who discontinued treatment on achieving remission (mainly patients with RA and PsA) had been taking the previous bDMARD for a longer mean time, as confirmed in the sensitivity analysis at 6 months. These results differ from those reported under conditions of clinical practice by Kádàr et al. [39] in a Hungarian cohort of patients with RA, AS, and PsA. The authors found an inverse association with a shorter time on treatment with bDMARDs, in contrast with other studies reporting results similar to ours [35, 40, 41].

The presence or absence of analytical biomarkers is one of the most reviewed factors in the literature and for which the most solid conclusions have been drawn, even if these are not definitive. Consistent with data published elsewhere, we found that patients with RA and a positive ACPA titer were less likely to discontinue treatment on achieving remission [7, 11, 25, 30, 42].

Our analysis revealed no association with disease activity. Moreover, the results of previous studies are contradictory in this regard. The heterogeneous nature of the measures used and the times in which they were made hamper comparison of the results and could explain many of the differences found [11, 13, 25, 28, 34].

In the multivariable models, we included those variables that were significant in the bivariable models and those that were clinically relevant. Unfortunately, we cannot provide information for some relevant variables, such as time in remission with treatment or tapering. While this information would be very useful in an analysis of the association with remission, the BIOBADASER registry evaluates safety and effectiveness and does not contain information on the aforementioned variables.

Our work is subject to a series of limitations. First, the concept of discontinuation due to the absence of disease activity as defined by the attending physician was considered a surrogate for remission. Although well-defined and more strict criteria have been proposed by ACR/EULAR, we believe that shared decision-making between physician and patient may be a more realistic goal in routine care [43]. Second, data are limited to patients who only received a first- or second-line b/tsDMARD in an effort to make our findings more robust and homogeneous, since remission is much more likely with first-line options. Third, the pooled analysis of 3 inflammatory diseases could hamper the initial interpretation; therefore, we performed a separate additional analysis. Our findings are also limited by the potential omission of variables related to remission due to the absence of these variables in our database. In addition, by including all patients who continue treatment and who discontinue for another reason as a comparator group, the control population may prove to be heterogeneous.

Our analysis made it possible to evaluate the prevalence of discontinuation in a cohort managed under conditions of daily clinical practice. Furthermore, in line with real-world evidence, discontinuation of bDMARDs in patients who achieve remission may be associated not only with the characteristics of the disease itself and the patient, but also with other factors, such as the patient and clinician’s perception, expectations, and experience.

## Conclusions

Discontinuation of bDMARDs on remission is uncommon in clinical practice. Smoking and longer disease duration are negative predictors of discontinuation, which could prove more difficult to achieve in patients requiring the addition of a csDMARD and more difficult to maintain over time in women.

Identifying profiles that help to better define patients who could discontinue treatment and maintain discontinuation would improve health outcomes in inflammatory diseases. Additional studies are necessary to provide more evidence on factors that predict the successful outcome of a discontinuation strategy in a real-world setting, especially including patients with AS and PsA.

### Supplementary Information

**Additional file 1: Supplementary Table 1.** Baseline characteristics of patients by disease. Footnote to supplementary table 1. Data are shown as mean (standard deviation), except for categorical variables, where they are expressed as n (%). RA: rheumatoid arthritis; AS: ankylosing spondylitis; PsA: psoriatic arthritis; bDMARD: biologic disease-modifying antirheumatic drug; csDMARD: conventional synthetic DMARD; tsDMARD: targeted synthetic DMARD; MTX: methotrexate, LFN: leflunomide; SSZ: sulfasalazine; i: inhibitor; RF: rheumatoid factor; ACPA: anti–citrullinated peptide antibody. *Moderate-high disease activity was defined as DAS28 ≥3.2 or BASDAI ≥4, depending on the disease.

**Additional file 2: Supplementary Table 2.** (2nd Sensitivity analysis). Demographic and clinical characteristics of patients with inflammatory arthritis who discontinued therapy according to clinical remission vs patients who continue. Footnote to table 1. Data are shown as mean (SD), except for categorical variables, which are shown as total number (percentage); Disc: Discontinuation; Rem: Remission; MTX: methotrexate, LFN: leflunomide; SSZ: sulfasalazine; i: inhibitor; RF: rheumatoid factor; ACPA: anti–citrullinated peptide antibody. *Moderate-high disease activity was defined as DAS28 ≥3.2 or BASDAI ≥4, depending on the disease.

**Additional file 3: Supplementary Table 3.** Sensitivity analysis 1: Clinical characteristics of patients who discontinued on achieving remission (data at 6 months after discontinuation). Footnote to supplementary table 2: P Rem: probability of remission/no remission at 6 months; Rem: remission; i: inhibitor; RA: rheumatoid arthritis; AS: ankylosing spondylitis; PsA: psoriatic arthritis; bDMARD: biologic disease-modifying antirheumatic drug; csDMARD: conventional synthetic DMARD; tsDMARD: targeted synthetic DMARD; MTX: methotrexate, LFN: leflunomide; SSZ: sulfasalazine; i: inhibitor; RF: rheumatoid factor; ACPA: anti–citrullinated peptide antibody. *Moderate-high disease activity was defined as DAS28 ≥3.2 or BASDAI ≥4, depending on the disease.

**Additional file 4: Supplementary Table 4.** Sensitivity analysis 1: Characteristics detailed by disease in patients who discontinued therapy on achieving remission (data at 6 months after discontinuation). Footnote to supplementary table 3: Disc.: Discontinuation; Rem: remission; b-DMARD: biologic-disease modifying antirheumatic drug; m: Months Rem: remission; RA: rheumatoid arthritis; AS: ankylosing spondylitis; PsA: psoriatic arthritis; csDMARD: conventional synthetic DMARD; MTX: methotrexate, LFN: leflunomide; SSZ: sulfasalazine; i: inhibitor; Abt: abatacept; RF: rheumatoid factor; ACPA: anti–citrullinated peptide antibody. *Moderate-high disease activity was defined as DAS28 ≥3.2 or BASDAI ≥4, depending on the disease.

**Additional file 5: Supplementary Table 5.** 2nd sensitivity analysis .Regression models for all the diseases together comparing patients who discontinued therapy according to clinical remission vs patients who continue. Footnote to table 2: OR: odds ratio. 95% CI: 95% confidence interval. TNF-i: tumor necrosis factor alfa inhibitor. RA: rheumatoid arthritis; AS: ankylosing spondylitis; PsA: psoriatic arthritis. csDMARD: conventional synthetic DMARD. * High activity: DAS28 ≥3.2 or BASDAI ≥4.

**Additional file 6: Supplementary Table 6.** 2nd Sensitivity analysis. Regression analysis by disease comparing patients who discontinued therapy according to clinical remission vs patients who continue. Footnote to Table 3: OR: odds ratio. 95% CI: 95% confidence interval. TNF-i: tumor necrosis factor alfa inhibitor. RA: rheumatoid arthritis; AS: ankylosing spondylitis; PsA: psoriatic arthritis: RF rheumatoid factor; ACPA: anti–citrullinated peptide antibody. Note: There were no ex-smokers for AS.

## Data Availability

The data that support the findings of this study are available from the Spanish Society of Rheumatology, although restrictions apply to the availability of these data, which were used under license for the current study and are therefore not publicly available. However, data are available from the authors upon reasonable request and with permission of the Spanish Society of Rheumatology.

## Abbreviations

RA: Rheumatoid arthritis
AS: Ankylosing spondylitis
PsA: Psoriatic arthritis
bDMARDs: Biological disease-modifying drugs
ACPA: Anti-citrullinated peptide antibody
tsDMARDs: Targeted synthetic DMARDs
RF: Rheumatoid factor
DAS28: 28-Joint Disease Activity Score
BASDAI: Bath Ankylosing Spondylitis Disease Activity Index
csDMARD: Conventional systemic DMARD
LFN: Leflunomide
SSZ: Sulfasalazine
i: Inhibitor
OR: Odds ratio
CI: Confidence interval
TNF-i: Tumor necrosis factor alfa inhibitor

## References

[1] Smolen JS, Aletaha D, Bijlsma JWJ, Breedveld FC, Boumpas D, Burmester G (2010). Treating rheumatoid arthritis to target: recommendations of an international task force. Ann Rheum Dis.

[2] Smolen JS, Braun J, Dougados M, Emery P, FitzGerald O, Helliwell P (2014). Treating spondyloarthritis, including ankylosing spondylitis and psoriatic arthritis, to target: recommendations of an international task force. Ann Rheum Dis.

[3] van der Heijde D, Ramiro S, Landewé R, Baraliakos X, Van den Bosch F, Sepriano A (2017). 2016 update of the ASAS-EULAR management recommendations for axial spondyloarthritis. Ann Rheum Dis.

[4] Smolen JS, Landewé RBM, Bijlsma JWJ, Burmester GR, Dougados M, Kerschbaumer A (2020). EULAR recommendations for the management of rheumatoid arthritis with synthetic and biological disease-modifying antirheumatic drugs: 2019 update. Ann Rheum Dis.

[5] Gossec L, Baraliakos X, Kerschbaumer A, de Wit M, McInnes I, Dougados M (2020). EULAR recommendations for the management of psoriatic arthritis with pharmacological therapies: 2019 update. Ann Rheum Dis.

[6] González-Álvaro I, Martínez-Fernández C, Dorantes-Calderón B, García-Vicuña R, Hernández-Cruz B, Herrero-Ambrosio A (2015). Spanish Rheumatology Society and Hospital Pharmacy Society Consensus on recommendations for biologics optimization in patients with rheumatoid arthritis, ankylosing spondylitis and psoriatic arthritis. Rheumatology.

[7] Verstappen M, van Mulligen E, de Jong PHP, van der Helm-Van Mil AHM (2020). DMARD-free remission as novel treatment target in rheumatoid arthritis: a systematic literature review of achievability and sustainability. RMD Open.

[8] Navarro-Compán V, Plasencia-Rodríguez C, de Miguel E, Balsa A, Martín-Mola E, Seoane-Mato D (2016). Anti-TNF discontinuation and tapering strategies in patients with axial spondyloarthritis: a systematic literature review. Rheumatology.

[9] Edwards CJ, Fautrel B, Schulze-Koops H, Huizinga TWJ, Kruger K (2017). Dosing down with biologic therapies: a systematic review and clinicians’ perspective. Rheumatology.

[10] Uhrenholt L, Christensen R, Dinesen WKH, Liboriussen CH, Andersen SS, Dreyer L (2022). Risk of flare after tapering or withdrawal of biologic/targeted synthetic disease-modifying anti-rheumatic drugs in patients with rheumatoid arthritis or axial spondyloarthritis: a systematic review and meta-analysis. Rheumatology (Oxford).

[11] Schlager L, Loiskandl M, Aletaha D, Radner H (2020). Predictors of successful discontinuation of biologic and targeted synthetic DMARDs in patients with rheumatoid arthritis in remission or low disease activity: a systematic literature review. Rheumatology.

[12] Michielsens CAJ, Boers N, den Broeder N, Wenink MH, van der Maas A, Mahler EAM (2020). Dose reduction and withdrawal strategy for TNF-inhibitors in psoriatic arthritis and axial spondyloarthritis: design of a pragmatic open-label, randomised, non-inferiority trial. Trials.

[13] Ruyssen-Witrand A, Rousseau V, Sommet A, Goupille P, Degboe Y, Constantin A (2022). Factors associated with drug-free remission at 5 year in early onset axial spondyloarthritis patients: data from the DESIR cohort. Joint Bone Spine.

[14] Baraliakos X, Kiltz U, Heldmann F, Sieper J, Braun J (2013). Withdrawal of biologic therapy in axial spondyloarthritis: the experience in established disease. Clin Exp Rheumatol.

[15] Gratacós J, Díaz del Campo Fontecha P, Fernández-Carballido C, JuanolaRoura X, Linares Ferrando LF, de Miguel Mendieta E (2018). Recomendaciones de la Sociedad Española de Reumatología sobre el uso de terapias biológicas en espondiloartritis axial. Reumatología Clínica.

[16] Landewé R, Sieper J, Mease P, Inman RD, Lambert RG, Deodhar A (2018). Efficacy and safety of continuing versus withdrawing adalimumab therapy in maintaining remission in patients with non-radiographic axial spondyloarthritis (ABILITY-3): a multicentre, randomised, double-blind study. The Lancet.

[17] Yoshida K, Radner H, Mjaavatten MD, Greenberg JD, Kavanaugh A, Kishimoto M (2015). Incidence and Predictors of Biological Antirheumatic Drug Discontinuation Attempts among Patients with Rheumatoid Arthritis in Remission: A CORRONA and NinJa Collaborative Cohort Study. J Rheumatol.

[18] Ebina K, Hashimoto M, Yamamoto W, Hirano T, Hara R, Katayama M (2019). Drug tolerability and reasons for discontinuation of seven biologics in 4466 treatment courses of rheumatoid arthritis—the ANSWER cohort study. Arthritis Res Ther.

[19] Martinez-Múgica C, Manso G (2020). Prescribing patterns and clinical outcomes of biological disease-modifying anti-rheumatic drugs for rheumatoid arthritis in Spain. Eur Rev Med Pharmacol Sci.

[20] Wetterslev M, Georgiadis S, Sørensen IJ, Pedersen SJ, Christiansen SN, Hetland ML (2022). Tapering of TNF inhibitors in axial spondyloarthritis in routine care — 2-year clinical and MRI outcomes and predictors of successful tapering. Rheumatology (Oxford).

[21] Bekele DI, Cheng E, Reimold A, Geier C, Ganuthula K, Walsh JA (2022). Tumor necrosis factor inhibitor (TNFi) persistence and reasons for discontinuation in a predominantly male cohort with axial spondyloarthritis. Rheumatol Int.

[22] Navarini L, Costa L, Tasso M, Chimenti MS, Currado D, Fonti GL (2020). Retention rates and identification of factors associated with anti-TNFα, anti-IL17, and anti-IL12/23R agents discontinuation in psoriatic arthritis patients: results from a real-world clinical setting. Clin Rheumatol.

[23] Naniwa T, Iwagaitsu S, Kajiura M (2020). Successful cessation of tumor necrosis factor inhibitor treatment in rheumatoid arthritis patients and potential predictors for early flare: An observational study in routine clinical care. Mod Rheumatol.

[24] van den Broek M, Klarenbeek NB, Dirven L, van Schaardenburg D, Hulsmans HMJ, Kerstens PJSM (2011). Discontinuation of infliximab and potential predictors of persistent low disease activity in patients with early rheumatoid arthritis and disease activity score-steered therapy: subanalysis of the BeSt study. Ann Rheum Dis.

[25] van der Woude D, Young A, Jayakumar K, Mertens BJ, Toes REM, van der Heijde D (2009). Prevalence of and predictive factors for sustained disease-modifying antirheumatic drug-free remission in rheumatoid arthritis: results from two large early arthritis cohorts. Arthritis Rheum.

[26] Wendling D, Guillot X, Gossec L, Prati C, Saraux A, Dougados M (2017). Remission is related to CRP and smoking in early axial spondyloarthritis. The DESIR cohort. Joint Bone Spine.

[27] Tweehuysen L, van den Ende CH, Beeren FMM, Been EMJ, van den Hoogen FHJ, den Broeder AA (2017). Little Evidence for Usefulness of Biomarkers for Predicting Successful Dose Reduction or Discontinuation of a Biologic Agent in Rheumatoid Arthritis: A Systematic Review: LITTLE EVIDENCE FOR PREDICTION OF BIOLOGIC TAPERING IN RA. Arthritis Rheumatol.

[28] Hamann P, Holland R, Hyrich K, Pauling JD, Shaddick G, Nightingale A (2017). Factors associated with sustained remission in rheumatoid arthritis in patients treated with anti-tumor necrosis factor. Arthritis Care Res (Hoboken).

[29] Saleem B, Keen H, Goeb V, Parmar R, Nizam S, Hensor EMA (2010). Patients with RA in remission on TNF blockers: when and in whom can TNF blocker therapy be stopped?. Ann Rheum Dis.

[30] van der Woude D, Visser K, Klarenbeek NB, Ronday HK, Peeters AJ, Kerstens PJSM (2012). Sustained drug-free remission in rheumatoid arthritis after DAS-driven or non-DAS-driven therapy: a comparison of two cohort studies. Rheumatology.

[31] Vittecoq O, Desouches S, Kozyreff M, Nicolau J, Pouplin S, Rottenberg P (2019). Relapse in rheumatoid arthritis patients undergoing dose reduction and withdrawal of biologics: are predictable factors more relevant than predictive parameters? An observational prospective real-life study. BMJ Open.

[32] Arnold S, Jaeger VK, Scherer A, Ciurea A, Walker UA, Kyburz D (2021). Discontinuation of biologic DMARDs in a real-world population of patients with rheumatoid arthritis in remission: outcome and risk factors. Rheumatology (Oxford).

[33] Hashimoto M, Furu M, Yamamoto W, Fujimura T, Hara R, Katayama M (2018). Factors associated with the achievement of biological disease-modifying antirheumatic drug-free remission in rheumatoid arthritis: the ANSWER cohort study. Arthritis Res Ther.

[34] Tanaka Y, Hirata S, Kubo S, Fukuyo S, Hanami K, Sawamukai N (2015). Discontinuation of adalimumab after achieving remission in patients with established rheumatoid arthritis: 1-year outcome of the HONOR study. Ann Rheum Dis.

[35] Burkard T, Williams RD, Vallejo-Yagüe E, Hügle T, Finckh A, Kyburz D (2021). Prediction of sustained biologic and targeted synthetic DMARD-free remission in rheumatoid arthritis patients. Rheumatol Adv Prac.

[36] Carron P, De Craemer AS, Renson T, Colman R, Elewaut D, Van den Bosch F (2021). TNFi-induced sustained clinical remission in peripheral spondyloarthritis patients cannot be maintained with a step-down strategy based on methotrexate. Rheumatology (Oxford).

[37] Maneiro JR, Souto A, Salgado E, Mera A, Gomez-Reino JJ (2015). Predictors of response to TNF antagonists in patients with ankylosing spondylitis and psoriatic arthritis: systematic review and meta-analysis. RMD Open.

[38] Nikiphorou E, Baraliakos X (2019). Treat to Target in Axial Spondyloarthritis. Rheumatic Disease Clinics of North America.

[39] Kádár G, Balázs E, Soós B, Laduver A, Keszthelyi P, Szekanecz Z (2014). Disease activity after the discontinuation of biological therapy in inflammatory rheumatic diseases. Clin Rheumatol.

[40] Brocq O, Millasseau E, Albert C, Grisot C, Flory P, Roux CH (2009). Effect of discontinuing TNFalpha antagonist therapy in patients with remission of rheumatoid arthritis. Joint Bone Spine.

[41] Almirall M, Salman-Monte TC, Lisbona MP, Maymó J (2015). Dose reduction of biological treatment in patients with axial spondyloarthritis in clinical remission: are there any differences between patients who relapsed and to those who remained in low disease activity?. Rheumatol Int.

[42] Klarenbeek NB, van der Kooij SM, Guler-Yuksel M, van Groenendael JHLM, Han KH, Kerstens PJSM (2011). Discontinuing treatment in patients with rheumatoid arthritis in sustained clinical remission: exploratory analyses from the BeSt study. Ann Rheum Dis.

[43] Felson DT, Smolen JS, Wells G, Zhang B, van Tuyl LH, Funovits J (2011). American College of Rheumatology/European League Against_Rheumatism provisional definition of remission in rheumatoid_arthritis for clinical trials. Ann Rheum Dis.

