# Correction: Factors associated with discontinuation of biologics in patients with inflammatory arthritis in remission: data from the BIOBADASER registry

**DOI:** 10.1186/s13075-023-03194-5

**Published:** 2023-10-25

**Authors:** Marta Valero, Carlos Sanchez-Piedra, Mercedes Freire, Maria Colazo, Noemi Busquets, Erardo Merino-Ibarra, Carlos Rodriguez-Lozano, Sara Manrique, Cristina Campos, Fernando Sanchez-Alonso, Isabel Castrejon

**Affiliations:** 1 Servicio de Reumatologia, Hospital Universitario Ramon Y CajalInstituto Ramon Y Cajal de Investigacion Sanitaria (IRYCIS), Carretera de Colmenar Viejo Km: 9,100, 28034 Madrid, Spain; 2 Health Technology Assessment Agency of Carlos III Institute of Health (ISCIII), Madrid, Spain; 3 Department of Rheumatology, Complejo Hospitalario Universitario A Coruna, A Coruna, Spain; 4 Department of Rheumatology, Hospital Universitario de Burgos, Burgos, Spain; 5 Department of Rheumatology, Hospital General de Granollers, Granollers, Barcelona, Spain; 6 Department of Rheumatology, Hospital Universitario Miguel Servet, Saragossa, Spain; 7 Department of Rheumatology, Hospital Universitario de Gran Canaria Doctor Negrin, Las Palmas de Gran Canaria, Spain; 8 Department of Rheumatology, Hospital Universitario Regional de Malaga (Hospital Carlos Haya), Malaga, Spain; 9 Department of Rheumatology, Hospital General Universitario de Valencia, Valencia, Spain; 10 Research Unit of Spanish Society of Rheumatology, Madrid, Spain; 11 Department of Rheumatology, Hospital General Universitario Gregorio MaranonInstituto de Investigacion Sanitaria Gregorio Maranon (IiSGM), Universidad Complutense de Madrid, Madrid, Spain

**Correction: Arthritis Res Ther 25, 86 (2023)**

**https://doi.org/10.1186/s13075-023-03045-3**

Following publication of the original article [1], the authors reported an error found in the abstract section wherein the word “shorter” should be replaced by “longer”. The correct sentence should reads:

The factors associated with a higher probability of discontinuation on remission were shorter disease duration (OR: 0.95; 95% CI: 0.91–0.99), no concomitant use of classic DMARDs (OR: 0.56; 95% CI: 0.34–0.92), and **longer** usage of the previous bDMARD (before the decision to discontinue biological therapy) (OR: 1.01; 95% CI: 1.01–1.02); in contrast, smoking status (OR: 2.48; 95% CI: 1.21–5.08) was associated with a lower probability.

The original article [1] has been updated.
