# Supplementary material for: Factors associated with discontinuation of biologics in patients with inflammatory arthritis in remission: data from the BIOBADASER registry
Source: Arthritis Res Ther. 2023 May 22;25:86. doi: 10.1186/s13075-023-03045-3 (PMC10201751; doi:10.1186/s13075-023-03045-3)
Supplement: Supplementary file 1 — Additional file 1: Supplementary Table 1. Baseline characteristics of patients by disease. Footnote to supplementary table 1. Data are shown as mean (standard deviation), except for categorical variables, where they are expressed as n (%). RA: rheumatoid arthritis; AS: ankylosing spondylitis; PsA: psoriatic arthritis; bDMARD: biologic disease-modifying antirheumatic drug; csDMARD: conventional synthetic DMARD; tsDMARD: targeted synthetic DMARD; MTX: methotrexate, LFN: leflunomide; SSZ: sulfasalazine; i: inhibitor; RF: rheumatoid factor; ACPA: anti–citrullinated peptide antibody. *Moderate-high disease activity was defined as DAS28 ≥3.2 or BASDAI ≥4, depending on the disease. [file 13075_2023_3045_MOESM1_ESM.docx]

**Supplementary table 1. Baseline characteristics of patients by disease**

|  | | RA | | | AS | | | PsA | | |
| --- | --- | --- | --- | --- | --- | --- | --- | --- | --- | --- |
|  | | Rem | No rem | P | Rem | No rem | P | Rem | No rem | P |
| No. of patients | | 30 | 1777 |  | 18 | 718 |  | 32 | 791 |  |
| Age (years), mean (SD) | | 58.9  (12.4) | 54.8  (13.0) | 0.086 | 36.8 (14.0) | 47.2 (12.7) | <0001 | 49.0 (12.4) | 49.4 (11.7) | 0.823 |
| Female sex, n (%) | | 26  (86.7) | 1412  (79.5) | 0.332 | 4  (22.2) | 235 (32.7) | 0.347 | 13 (40.6) | 443 (56.0) | 0.086 |
| Age at diagnosis, mean (SD) | | 52.3  (11.4) | 45.7 (13.7) | 0.009 | 32.0 (12.3) | 37.9 (13.4) | 0.063 | 41.3 (12.9) | 42.5 (12.3) | 0.615 |
| Disease duration, mean (SD) | | 6.6  (3.8) | 9.0  (8.4) | 0.113 | 4.8 (6.4) | 9.3  (9.9) | 0.057 | 7.6  (4.2) | 7.0  (6.7) | 0.589 |
| Smoking, n and % | Non-smoker | 27  (90.0) | 1235 (69.5) | 0.113 | 14  (77.8) | 406 (56.5) | 0.224 | 27 (84.4) | 524 (66.2) | 0.159 |
|  | Current | 2  (6.7) | 318  (17.9) |  | 3  (16.7) | 225  (31.3) |  | 4  (12.5) | 152 (19.2) |  |
|  | Ex-smoker | 1 (3.3) | 185 (10.4) |  | 0 (0) | 62 (8.6) |  | 1 (3.1) | 82(10.4) |  |
| Charlson Comorbidity Index, mean (SD) | | 2.2 (1.7) | 2.3 (1.6) | 0.951 | 1.2 (0.5) | 1.8 (1.3) | 0.033 | 1.8(1.7) | 1.9 (1.2) | 0.847 |
| Previous biologic, n (%) | First-line | 15 (50.0) | 987 (55.5) | 0.545 | 14 (77.8) | 443 (61.7) | 0.165 | 18 (56.3) | 478 (60.4) | 0.636 |
|  | Second-line | 15 (50.0) | 790 (44.5) |  | 4 (22.2) | 275 (38.3) |  | 14 (43.8) | 313 (39.6) |  |
| Concomitant csDMARD, n (%) | MTX | 13 (56.5) | 934 (72.3) | 0.095 | 1  (9.1) | 115 (29.8) | 0.137 | 11 (50.0) | 340 (70.0) | 0.048 |
|  | LFN | 7 (35.0) | 460 (41.7) | 0.547 | 0(0) | 18 (5.4) | 0.430 | 4 (21.1) | 150 (38.0) | 0.136 |
|  | SSZ | 1 (5.3) | 58 (6.4) | 0.837 | 4 (33.3) | 74 (20.8) | 0.298 | 0 (0) | 37 (10.9) | 0.128 |
| Time on previous bDMARD, mean (SD) | | 59.3  (49.6) | 23.3  (34.1) | <0.001 | 35.5  (23.0) | 27.0 (32.8) | 0.275 | 49.9 (35.8) | 23.9 (34.3) | <0.001 |
| Treatment at discontinuation, n (%) | TNF-i | 22 (73.3) | 1195 (67.2) | 0.903 | 18 (100) | 664 (92.5) | 0.691 | 32 (100) | 644 (81.4) | 0.299 |
|  | IL6-i | 3 (10.0) | 193 (10.9) |  | 0 (0) | 1 (0.1) |  | 0 (0) | 0 (0) |  |
|  | CD20-i | 2 (6.7) | 187 (10.5) |  | 0 (0) | 0 (0) |  | 0 (0) | 0 (0) |  |
|  | JAK-i | 0 (0) | 68 (3.8) |  | 0 (0) | 0 (0) |  | 0 (0) | 2 (0.3) |  |
|  | IL17-i | 0 (0) | 0 (0) |  | 0 (0) | 52 (7.2) |  | 0 (0) | 57 (7.2) |  |
|  | IL12-23-i | 0 (0) | 0 (0) |  | 0 (0) | 1 (0.1) |  | 0 (0) | 27 (3.4) |  |
|  | PDE4-i | 0 (0) | 0 (0) |  | 0 (0) | 0 (0) |  | 0 (0) | 57 (7.2) |  |
|  | Abatacept | 3 (10.0) | 128 (7.2) |  | 0 (0) | 0 (0) |  | 0 (0) | 3 (0.4) |  |
| RF-positive, n (%) | | 9 (30.0) | 772 (43.4) | 0.424 | - |  | ~~-~~ |  |  | ~~-~~ |
| ACPA-positive, n (%) | | 3 (23.1) | 729 (72.0) | <0.001 | - |  | ~~-~~ |  |  | ~~-~~ |
| HLA-B27–positive, n (%) | | - | - | - | 12 (66.7) | 517 (72.0) | 0.883 | 6 (18.8) | 110 (13.9) | 0.549 |
| Moderate-high activity at initiation of biologic * | | 2 (10.0) | 208 (16.3) | 0.446 | 3 (25.0) | 113 (18.8) | 0.589 | 4 (19.1) | 127 (19.8) | 0.931 |

Footnote to supplementary table 1. Data are shown as mean (standard deviation), except for categorical variables, where they are expressed as n (%).

RA: rheumatoid arthritis; AS: ankylosing spondylitis; PsA: psoriatic arthritis; bDMARD: biologic disease-modifying antirheumatic drug; csDMARD: conventional synthetic DMARD; tsDMARD: targeted synthetic DMARD; MTX: methotrexate, LFN: leflunomide; SSZ: sulfasalazine; i: inhibitor; RF: rheumatoid factor; ACPA: anti–citrullinated peptide antibody.

*Moderate-high disease activity was defined as DAS28 ≥3.2 or BASDAI ≥4, depending on the disease.
