# Supplementary material for: Factors associated with discontinuation of biologics in patients with inflammatory arthritis in remission: data from the BIOBADASER registry
Source: Arthritis Res Ther. 2023 May 22;25:86. doi: 10.1186/s13075-023-03045-3 (PMC10201751; doi:10.1186/s13075-023-03045-3)
Supplement: Supplementary file 2 — Additional file 2: Supplementary Table 2. (2nd Sensitivity analysis). Demographic and clinical characteristics of patients with inflammatory arthritis who discontinued therapy according to clinical remission vs patients who continue. Footnote to table 1. Data are shown as mean (SD), except for categorical variables, which are shown as total number (percentage); Disc: Discontinuation; Rem: Remission; MTX: methotrexate, LFN: leflunomide; SSZ: sulfasalazine; i: inhibitor; RF: rheumatoid factor; ACPA: anti–citrullinated peptide antibody. *Moderate-high disease activity was defined as DAS28 ≥3.2 or BASDAI ≥4, depending on the disease. [file 13075_2023_3045_MOESM2_ESM.docx]

**Supplementary table 2. (2º Sensitivity analysis)**. **Demographic and clinical characteristics of patients with inflammatory arthritis who discontinued therapy according to clinical remission vs patients who continue**

|  | | Total | Disc.  owing to  rem | No disc.  owing to  rem | P value |
| --- | --- | --- | --- | --- | --- |
| N | | 1748 | 80 | 1668 |  |
| Median duration of follow-up (years)  (IQR:p25-p75) | | 3,00 (1.00-6.90) | 5.57  (3.73-8.89) | 2.70  (1.00-6.60) | <0.001 |
| Age, years mean (SD) | | 52.7 (13.8) | 49.9 (15.1) | 52.8 (13.8) | 0.07 |
| Female sex, n (%) | | 1105 (63.2) | 43.0 (53.8) | 1062 (63.7) | 0.072 |
| Age at diagnosis, mean (SD) | | 43.4 (14.1) | 43.3 (14.4) | 43.4 (14.1) | 0.977 |
| Disease duration, years, mean (SD) | | 8.2 (8.3) | 6.6 (4.7) | 8.3 (8.3) | 0.005 |
| Smoker, n (%) | Never | 1155 (66.1) | 68 (85.0) | 1087 (65.2) | 0.003 |
|  | Current | 368 (21.1) | 9 (11.3) | 359 (21.5) |  |
|  | Previous | 175 (10.0) | 2 (2.5) | 173 (10.4) |  |
| Charlson Comorbidity Index, mean (SD) | | 2.2 (1.5) | 1.8 (1.5) | 2.2 (1.5) | 0.043 |
| Previous biologic DMARD, n (%) | First-line | 1041 (59.6) | 47.0 (58.8) | 994 (59.6) | 0.881 |
|  | Second-line | 707 (40.4) | 33.0 (41.3) | 674 (40.4) |  |
| Concomitant DMARD | MTX | 695 (61.3) | 25.0 (44.6) | 670 (62.2) | 0.008 |
|  | LFN | 330 (34.3) | 11.0 (22.0) | 319 (35.0) | 0.059 |
|  | SSZ | 96 (11.5) | 5.0 (10.0) | 91 (11.6) | 0.734 |
| Time on treatment with the previous biologic agent, mean (SD) | | 26.2 (37.4) | 50.2 (40.0) | 25.0 (36.9) | <0.001 |
| Biologic discontinued, n (%) | TNF-i | 1270 (72.7) | 72 (90.0) | 1198 (71.8) | 0.088 |
|  | IL6-i | 124 (7.1) | 3 (3.8) | 121 (7.3) |  |
|  | CD20-i | 182 (10.4) | 2 (2.5) | 180 (10.8) |  |
|  | JAK-i | 39 (2.2) | 0 (0) | 39 (2.3) |  |
|  | IL17-i | 36 (2.1) | 0 (0) | 36 (2.2) |  |
|  | IL12-23-i | 6 (0.3) | 0 (0) | 6 (0.4) |  |
|  | Apremilast | 24 (1.4) | 0 (0) | 24 (1.4) |  |
|  | Abatacept | 62 (3.5) | 3 (3.8) | 59 (3.5) |  |
| RF-positive, n (%) | | 473 (27.1) | 9 (11.3) | 464 (27.8) | 0.043 |
| ACPA-positive, n (%) | | 437 (41.9) | 3 (8.6) | 434 (43.1) | <0.001 |
| HLA-B27–positive, n (%) | | 375 (21.5) | 18 (22.5) | 357 (21.4) | 0.030 |
| Moderate-high disease activity at initiation of biologics * | | 310 (23.3) | 9 (17.0) | 301 (23.6) | 0.256 |

Footnote to table 1. Data are shown as mean (SD), except for categorical variables, which are shown as total number (percentage); Disc: Discontinuation; Rem: Remission; MTX: methotrexate, LFN: leflunomide; SSZ: sulfasalazine; i: inhibitor; RF: rheumatoid factor; ACPA: anti–citrullinated peptide antibody.

*Moderate-high disease activity was defined as DAS28 ≥3.2 or BASDAI ≥4, depending on the disease.
