# Supplementary material for: Factors associated with discontinuation of biologics in patients with inflammatory arthritis in remission: data from the BIOBADASER registry
Source: Arthritis Res Ther. 2023 May 22;25:86. doi: 10.1186/s13075-023-03045-3 (PMC10201751; doi:10.1186/s13075-023-03045-3)
Supplement: Supplementary file 3 — Additional file 3: Supplementary Table 3. Sensitivity analysis 1: Clinical characteristics of patients who discontinued on achieving remission (data at 6 months after discontinuation). Footnote to supplementary table 2: P Rem: probability of remission/no remission at 6 months; Rem: remission; i: inhibitor; RA: rheumatoid arthritis; AS: ankylosing spondylitis; PsA: psoriatic arthritis; bDMARD: biologic disease-modifying antirheumatic drug; csDMARD: conventional synthetic DMARD; tsDMARD: targeted synthetic DMARD; MTX: methotrexate, LFN: leflunomide; SSZ: sulfasalazine; i: inhibitor; RF: rheumatoid factor; ACPA: anti–citrullinated peptide antibody. *Moderate-high disease activity was defined as DAS28 ≥3.2 or BASDAI ≥4, depending on the disease. [file 13075_2023_3045_MOESM3_ESM.docx]

**Supplementary table 3. First Sensitivity analysis: Clinical characteristics of patients who discontinued on achieving remission (data at 6 months after discontinuation)**

| **Variable** | | No disc. due to remission | bDMARD-free  6 months | All | p Rem |
| --- | --- | --- | --- | --- | --- |
| **N** | | 3286 | 53 | 3366 |  |
| **Age (years)** | | 51.8 (13.0) | 50.4 (16.5) | 51.8 (13.1) | 0.425 |
| **Female sex** | | 2090 (63.6) | 27 (50.9) | 2133 (63.4) | 0.058 |
| **Age at diagnosis** | | 43.2 (13.6) | 44.1 (15.3) | 43.2 (13.7) | 0.663 |
| **Disease duration** | | 8.6 (8.4) | 6.3 (4.1) | 8.6 (8.4) | 0.051 |
| **Smoking** | **Current** | 2165 (65.9) | 47 (88.7) | 2233 (66.3) | 0.006 |
|  | **Ex-smoker** | 695 (21.2) | 4 (7.5) | 704 (20.9) |  |
|  | **Never** | 329 (10.0) | 1 (1.9) | 331 (9.8) |  |
| **Charlson Comorbidity Index** | | 2.1 (1.4) | 2.0 (1.8) | 2.1 (1.4) | 0.677 |
| **Previous bDMARD** | **First-line** | 1908 (58.1) | 30 (56.6) | 1955 (58.1) | 0.831 |
|  | **Second-line** | 1378 (41.9) | 23 (43.4) | 1411 (41.9) |  |
| **Corticosteroids** | | 1442 (63.9) | 21 (55.3) | 1470 (63.5) | 0.271 |
| **Concomitant csDMARD** | **MTX** | 1389 (64.2) | 18 (48.6) | 1414 (63.7) | 0.051 |
|  | **LFN** | 628 (34.2) | 11 (33.3) | 639 (33.9) | 0.913 |
|  | **SSZ** | 169 (10.6) | 4 (12.1) | 174 (10.6) | 0.778 |
| **Mean time until previous bDMARD discontinued** | | 24.3 (33.9) | 48.3 (39.5) | 24.9 (34.3) | <0.001 |
| **Treatment at discontinuation** | **TNF-i** | 2503 (76.2) | 45 (84.9) | 2575 (76.5) | 0.779 |
|  | **IL6-i** | 194 (5.9) | 3 (5.7) | 197 (5.9) |  |
|  | **CD20-i** | 187 (5.7) | 2 (3.8) | 189 (5.6) |  |
|  | **JAK-i** | 70 (2.1) | 0 (0.0) | 70 (2.1) |  |
|  | **IL1-i** | 5 (0.2) | 0 (0.0) | 5 (0.1) |  |
|  | **IL17A-i** | 109 (3.3) | 0 (0.0) | 109 (3.2) |  |
|  | **IL12/23-i** | 28 (0.9) | 0 (0.0) | 28 (0.8) |  |
|  | **PDE4-i** | 57 (1.7) | 0 (0.0) | 57 (1.7) |  |
|  | **Abatacept** | 131 (4.0) | 3 (5.7) | 134 (4.0) |  |
| **RF** | | 819 (24.9) | 7 (13.2) | 828 (24.6) | 0.209 |
| **ACPA** | | 755 (37.3) | 3 (12.5) | 758 (36.8) | 0.029 |
| **HLA-B27** | | 653 (19.9) | 10 (18.9) | 671 (19.9) | 0.163 |
| **Moderate-high activity*** | | 448 (17.8) | 3 (7.7) | 457 (17.8) | 0.100 |

F**ootnote to supplementary table 2**: Disc: Distontinuation. P Rem: probability of remission/no remission at 6 months; Rem: remission; i: inhibitor; RA: rheumatoid arthritis; AS: ankylosing spondylitis; PsA: psoriatic arthritis; bDMARD: biologic disease-modifying antirheumatic drug; csDMARD: conventional synthetic DMARD; tsDMARD: targeted synthetic DMARD; MTX: methotrexate, LFN: leflunomide; SSZ: sulfasalazine; i: inhibitor; RF: rheumatoid factor; ACPA: anti–citrullinated peptide antibody.

*Moderate-high disease activity was defined as DAS28 ≥3.2 or BASDAI ≥4, depending on the disease.
