# Supplementary material for: Factors associated with discontinuation of biologics in patients with inflammatory arthritis in remission: data from the BIOBADASER registry
Source: Arthritis Res Ther. 2023 May 22;25:86. doi: 10.1186/s13075-023-03045-3 (PMC10201751; doi:10.1186/s13075-023-03045-3)
Supplement: Supplementary file 4 — Additional file 4: Supplementary Table 4. Sensitivity analysis 1: Characteristics detailed by disease in patients who discontinued therapy on achieving remission (data at 6 months after discontinuation). Footnote to supplementary table 3: Disc.: Discontinuation; Rem: remission; b-DMARD: biologic-disease modifying antirheumatic drug; m: Months Rem: remission; RA: rheumatoid arthritis; AS: ankylosing spondylitis; PsA: psoriatic arthritis; csDMARD: conventional synthetic DMARD; MTX: methotrexate, LFN: leflunomide; SSZ: sulfasalazine; i: inhibitor; Abt: abatacept; RF: rheumatoid factor; ACPA: anti–citrullinated peptide antibody. *Moderate-high disease activity was defined as DAS28 ≥3.2 or BASDAI ≥4, depending on the disease. [file 13075_2023_3045_MOESM4_ESM.docx]

**Supplementary table 4. First Sensitivity analysis: Characteristics detailed by disease in patients who discontinued therapy on achieving remission (data at 6 months after discontinuation)**

|  | | RA | | | | AS | | | | PsA | | | |
| --- | --- | --- | --- | --- | --- | --- | --- | --- | --- | --- | --- | --- | --- |
| **Variable** | | No disc. due to rem. | b-DMARD free 6 m | All | P | No disc. due to rem. | b-DMARD free 6 m | All | P | No disc. due to rem. | b-DMARD free 6 m | All | P |
| **N** | | 1777 | 22 | 1807 |  | 718 | 12 | 736 |  | 791 | 19 | 823 |  |
| **Age (years)** | | 54.8 (13.0) | 59.8 (13.1) | 54.8 (13.0) | 0.072 | 47.2 (12.7) | 33.7  (13.5) | 46.9  (12.9) | <0.001 | 49.4  (11.7) | 50.0  (13.5) | 49.4  (11.7) | 0.836 |
| **Female sex** | | 1412 (79.5) | 19  (86.4) | 1438 (79.6) | 0.425 | 235 (32.7) | 3  (25.0) | 239  (32.5) | 0.571 | 443  (56.0) | 5  (26.3) | 456  (55.4) | 0.010 |
| **Age at diagnosis** | | 45.7 (13.7) | 53.0  (11.4) | 45.8 (13.7) | 0.013 | 37.9 (13.4) | 31.3  (13.5) | 37.8  (13.4) | 0.091 | 42.5  (12.3) | 41.8  (14.2) | 42.4  (12.3) | 0.807 |
| **Disease duration** | | 9.0  (8.4) | 6.8  (3.7) | 9.0  (8.4) | 0.209 | 9.3  (9.9) | 2.4  (2.5) | 9.2  (9.8) | 0.017 | 7.0  (6.7) | 8.2  (3.8) | 7.0  (6.6) | 0.411 |
| **Smoking** | **Current** | 1235 (69.5) | 20 (90.9) | 1262 (69.8) | 0.161 | 406 (56.5) | 10  (83.3) | 420  (57.1) | 0.150 | 524  (66.2) | 17  (89.5) | 551  (67.0) | 0.197 |
|  | **Ex-smoker** | 318 (17.9) | 2  (9.1) | 320 (17.7) |  | 225 (31.3) | 1  (8.3) | 228  (31.0) |  | 152  (19.2) | 1  (5.3) | 156  (19.0) |  |
|  | **Never** | 185 (10.4) | 0  (0.0) | 186 (10.3) |  | 62  (8.6) | 0  (0.0) | 62  (8.4) |  | 82  (10.4) | 1  (5.3) | 83  (10.1) |  |
| **Charlson Comorbidity Index** | | 2.3  (1.6) | 2.5 (1.8) | 2.3  (1.6) | 0.459 | 1.8  (1.3) | 1.0  (0.0) | 1.8  (1.3) | 0.03 | 1.9  (1.2) | 2.0  (2.2) | 1.9  (1.2) | 0.607 |
| **Previous bDMARD** | **First-line** | 987 (55.5) | 10 (45.5) | 1002 (55.5) | 0.344 | 443 (61.7) | 9  (75.0) | 457  (62.1) | 0.347 | 478  (60.4) | 11  (57.9) | 496  (60.3) | 0.823 |
|  | **Second-line** | 790 (44.5) | 12 (54.5) | 805 (44.5) |  | 275 (38.3) | 3  (25.0) | 279  (37.9) |  | 313  (39.6) | 8  (42.1) | 327 (39.7) |  |
| **Corticosteroids** | | 1119 (78.6) | 14 (77.8) | 1136 (78.5) | 0.934 | 82  (22.6) | 1  (14.3) | 83  (22.2) | 0.602 | 241  (51.4) | 6  (46.2) | 251 (51.0) | 0.710 |
| **Concomitant csDMARD** | **MTX** | 934 (72.3) | 9  (56.3) | 947 (72.0) | 0.155 | 115 (29.8) | 1  (14.3) | 116  (29.2) | 0.373 | 340  (70.0) | 8  (57.1) | 351 (69.1) | 0.304 |
|  | **LFN** | 460 (41.7) | 7  (50.0) | 467 (41.6) | 0.532 | 18  (5.4) | 0  (0.0) | 18  (5.2) | 0.529 | 150  (38.0) | 4 (33.3) | 154 (37.2) | 0.744 |
|  | **SSZ** | 58  (6.4) | 1  (7.7) | 59  (6.4) | 0.854 | 74  (20.8) | 3 (37.5) | 78  (21.3) | 0.254 | 37  (10.9) | 0  (0.0) | 37  (10.4) | 0.226 |
| **Mean time since discontinuation of previous bDMARD** | | 23.3 (34.1) | 54.3 (48.6) | 23.9 (34.7) | <0.001 | 27.0 (32.8) | 28.4  (22.4) | 27.2  (32.6) | 0.879 | 23.9  (34.3) | 54.0 (33.4) | 24.9 (34.7) | <0.001 |
| **Treatment at discontinuation** | **TNF-i** | 1195 (67.2) | 14  (63.6) | 1217 (67.3) | 0.881 | 664 (92.5) | 12  (100.) | 682  (92.7) | 0.807 | 644  (81.4) | 19  (100.) | 676 (82.1) | 0.634 |
|  | **IL6-i** | 193 (10.9) | 3  (13.6) | 196 (10.8) |  | 1  (0.1) | 0  (0.0) | 1  (0.1) |  | 0  (0.0) | 0  (0.0) | 0  (0.0) |  |
|  | **CD20-i** | 187 (10.5) | 2  (9.1) | 189 (10.5) |  | 0  (0.0) | 0  (0.0) | 0  (0.0) |  | 0  (0.0) | 0  (0.0) | 0  (0.0) |  |
|  | **JAK-i** | 68  (3.8) | 0  (0.0) | 68  (3.8) |  | 0  (0.0) | 0  (0.0) | 0  (0.0) |  | 2  (0.3) | 0  (0.0) | 2  (0.2) |  |
|  | **IL1-i** | 4 (0.2) | 0 (0.0) | 4 (0.2) |  | 0 (0.0) | 0 (0.0) | 0 (0.0) |  | 1 (0.1) | 0 (0.0) | 1 (0.1) |  |
|  | **IL17A-i** | 0 (0.0) | 0 (0.0) | 0 (0.0) |  | 52 (7.2) | 0 (0.0) | 52 (7.1) |  | 57 (7.2) | 0 (0.0) | 57 (6.9) |  |
|  | **IL12/23-i** | 0 (0.0) | 0 (0.0) | 0 (0.0) |  | 1 (0.1) | 0 (0.0) | 1 (0.1) |  | 27 (3.4) | 0 (0.0) | 27 (3.3) |  |
|  | **PDE4-i** | 0 (0.0) | 0 (0.0) | 0 (0.0) |  | 0 (0.0) | 0 (0.0) | 0 (0.0) |  | 57 (7.2) | 0 (0.0) | 57 (6.9) |  |
|  | **Abt** | 128(7.2) | 3 (13.6) | 131 (7.2) |  | 0 (0.0) | 0 (0.0) | 0 (0.0) |  | 3 (0.4) | 0 (0.0) | 3 (0.4) |  |
| **RF** | | 772 (43.4) | 7  (31.8) | 781 (43.2) | 0.285 | - | - | - |  | - | - | - |  |
| **ACPA** | | 729 (72.0) | 3  (27.3) | 732 (71.4) | 0.004 | - | - | - |  | - | - | - |  |
| **HLA-B27** | | - | - | - |  | 517 (72.0) | 7  (58.3) | 529  (71.9) | 0.546 | 110  (13.9) | 3  (15.8) | 116 (14.1) | 0.935 |
| **Moderate-high activity*** | | 208  (16.3) | 1  (6.3) | 210 (16.2) | 0.277 | 113 (18.8) | 2  (25.0) | 116  (19.0) | 0.658 | 127  (19.8) | 0  (0.0) | 131 (19.8) | 0.055 |

**Footnote to supplementary table 3.** Disc.: Discontinuation; Rem: remission; b-DMARD: biologic-disease modifying antirheumatic drug; m: Months; RA: rheumatoid arthritis; AS: ankylosing spondylitis; PsA: psoriatic arthritis; csDMARD: conventional synthetic DMARD; MTX: methotrexate, LFN: leflunomide; SSZ: sulfasalazine; i: inhibitor; Abt: abatacept; RF: rheumatoid factor; ACPA: anti–citrullinated peptide antibody.

*Moderate-high disease activity was defined as DAS28 ≥3.2 or BASDAI ≥4, depending on the disease.
