# Supplementary material for: Factors associated with discontinuation of biologics in patients with inflammatory arthritis in remission: data from the BIOBADASER registry
Source: Arthritis Res Ther. 2023 May 22;25:86. doi: 10.1186/s13075-023-03045-3 (PMC10201751; doi:10.1186/s13075-023-03045-3)
Supplement: Supplementary file 5 — Additional file 5: Supplementary Table 5. 2nd sensitivity analysis .Regression models for all the diseases together comparing patients who discontinued therapy according to clinical remission vs patients who continue. Footnote to table 2: OR: odds ratio. 95% CI: 95% confidence interval. TNF-i: tumor necrosis factor alfa inhibitor. RA: rheumatoid arthritis; AS: ankylosing spondylitis; PsA: psoriatic arthritis. csDMARD: conventional synthetic DMARD. * High activity: DAS28 ≥3.2 or BASDAI ≥4. [file 13075_2023_3045_MOESM5_ESM.docx]

**Supplementary Table 5. 2ª sensitivity analysis. Regression models for all the diseases together comparing patients who discontinued therapy according to clinical remission vs patients who continue**

|  | | Bivariable model | | | Multivariable model | | |
| --- | --- | --- | --- | --- | --- | --- | --- |
|  | | Crude OR | 95% CI | P value | Adjusted OR | 95% CI | P value |
| Female sex | | 0.66 | (0.42-1.04) | 0.074 | 0.73 | (0.43-1.25) | 0.255 |
| Age at onset | | 0.99 | (0.97-1.00) | 0.071 | 1.01 | (0.99-1.03) | 0.482 |
| Disease (ref RA) | AS | 1.53 | (0.84-2.78) | 0.161 | 1.15 | (0.53-2.48) | 0.731 |
|  | PsA | 3.04 | (1.82-5.08) | <0.001 | 2.23 | (1.22-4.10) | 0.010 |
| TNF-i (ref remaining treatments) | | 3.53 | (1.69-7.39) | 0.001 | 1.64 | (0.70-3.86) | 0.259 |
| Smoking (ref smoker) | Non-smoker | 2.50 | (1.23-5.05) | 0.011 | 2.62 | (1.27-5.42) | 0.009 |
|  | Ex-smoker | 0.46 | (0.10-2.16) | 0.325 | 0.62 | (0.13-2.97) | 0.550 |
| Second line of treatment (vs first line) | | 1.04 | (0.66-1.63) | 0.881 | 1.71 | (1.03-2.84) | 0.038 |
| Disease duration | | 0.95 | (0.92-0.99) | 0.006 | 0.94 | (0.90-0.98) | 0.002 |
| Concomitant csDMARD | | 0.65 | (0.41-1.01) | 0.057 | 0.60 | (0.37-1.00) | 0.049 |
| Moderate-high activity* | | 0.66 | (0.32-1.37) | 0.268 |  |  |  |
| Time on treatment with the previous biologic agent | | 1.01 | (1.01-1.02) | <0.001 | 1.01 | (1.01-1.02) | <0.001 |
| Year of discontinuation of treatment | | 0.97 | (0.93-1.02) | 0.277 | 0.97 | (0.91-1.02) | 0.235 |

Footnote to table 2: OR: odds ratio. 95% CI: 95% confidence interval. TNF-i: tumor necrosis factor alfa inhibitor. RA: rheumatoid arthritis; AS: ankylosing spondylitis; PsA: psoriatic arthritis. csDMARD: conventional synthetic DMARD.

* High activity: DAS28 ≥3.2 or BASDAI ≥4
