# Supplementary material for: Factors associated with discontinuation of biologics in patients with inflammatory arthritis in remission: data from the BIOBADASER registry
Source: Arthritis Res Ther. 2023 May 22;25:86. doi: 10.1186/s13075-023-03045-3 (PMC10201751; doi:10.1186/s13075-023-03045-3)
Supplement: Supplementary file 6 — Additional file 6: Supplementary Table 6. 2nd Sensitivity analysis. Regression analysis by disease comparing patients who discontinued therapy according to clinical remission vs patients who continue. Footnote to Table 3: OR: odds ratio. 95% CI: 95% confidence interval. TNF-i: tumor necrosis factor alfa inhibitor. RA: rheumatoid arthritis; AS: ankylosing spondylitis; PsA: psoriatic arthritis: RF rheumatoid factor; ACPA: anti–citrullinated peptide antibody. Note: There were no ex-smokers for AS. [file 13075_2023_3045_MOESM6_ESM.docx]

**Supplementary table 6. 2º Sensitivity analysis. Regression analysis by disease comparing patients who discontinued therapy according to clinical remission vs patients who continue**

|  | | | RA | | | AS | | | PsA | | |
| --- | --- | --- | --- | --- | --- | --- | --- | --- | --- | --- | --- |
|  | | | OR | 95% CI | P value | OR | 95% CI | P value | OR | 95% CI | P value |
| Female sex | | | 1.23 | (0.12-12.22) | 0.861 | 0.45 | (0.14-1.48) | 0.189 | 0.68 | (0.31-1.50) | 0.339 |
| Age | | | 1.01 | (0.96-1.06) | 0.678 | 0.96 | (0.92-1.00) | 0.046 | 1.00 | (0.97-1.04) | 0.932 |
| TNF-i | | | 0.71 | (0.14-3.54) | 0.675 | - | - | - | - | - | - |
| Smoking (ref smoker) | | Non-smoker | 2.75 | (0.29-26.10) | 0.378 | 2.54 | (0.68-9.47) | 0.166 | 2.01 | (0.65-6.23) | 0.224 |
|  |  | Ex-smoker | 1.17 | (0.06-22.96) | 0.916 | - | - | - | 0.61 | (0.06-5.92) | 0.668 |
| Order of treatment | | | 4.96 | (1.16-21.20) | 0.031 | 1.02 | (0.30-3.48) | 0.974 | 1.96 | (0.85-4.52) | 0.113 |
| Disease duration | | | 0.97 | (0.88-1.06) | 0.471 | 0.93 | (0.85-1.03) | 0.150 | 0.98 | (0.92-1.04) | 0.523 |
| ACPA  (ref negative) | Positive | | 0.11 | (0.02-0.51) | 0.005 | - | - | - | - | - | - |
| HLA B27  (ref negative) | Positive | | - | - | - | 0.38 | (0.11-1.37) | 0.140 | 1.30 | (0.42-4.01) | 0.646 |
| Time on treatment with the previous biologic agent |  | | 1.03 | (1.01-1.05) | 0.001 | - | - | - | 1.01 | (1.00-1.02) | 0.011 |
| Year of discontinuation of treatment |  | | 0.87 | (0.71-1.05) | 0.144 | 0.90 | (0.80-1.02) | 0.109 | 0.99 | (0.90-1.09) | 0.840 |

Footnote to Table 3: OR: odds ratio. 95% CI: 95% confidence interval. TNF-i: tumor necrosis factor alfa inhibitor. RA: rheumatoid arthritis; AS: ankylosing spondylitis; PsA: psoriatic arthritis: RF rheumatoid factor; ACPA: anti–citrullinated peptide antibody.

Note: There were no ex-smokers for AS.
